# Comparison of the biomechanical effects of the post-core crown, endocrown and inlay crown after deep margin elevation and its clinical significance

**DOI:** 10.1186/s12903-024-04604-z

**Published:** 2024-08-23

**Authors:** Feng Wu, Xiaomin Su, Yue Shi, Juan Bai, Jing Feng, Xilin Sun, Xuanqi Wang, Hongyan Wang, Jiayu Wen, Jie Kang

**Affiliations:** Shanxi Medical University School and Hospital of Stomatology, Taiyuan, China

**Keywords:** Post core crown, Endocrown, Inlay crown, Deep margin elevation, Finite element analysis

## Abstract

**Background:**

The purpose of this in vitro study was to compare and evaluate the stress distribution of maxillary first premolar residual crowns restored with post-core crowns, endocrowns and inlay crowns after deep margin elevation, to explore the fitting restoration for residual crowns using finite element analysis.

**Methods:**

A healthy complete right maxillary first premolar from a male adult was scanned by cone beam computed tomography (CBCT). The finite element model of the tooth was established by reverse engineering software such as Mimics, Geomagic and Hypermesh. On this basis, the residual crown model after deep margin elevation was made, and the experimental group models were divided into three groups, those restored with post core crowns, endocrowns and inlay crowns. Vertical and oblique static loads were applied to the experimental models to simulate the force on the tooth during mastication (the loading position was located in the central fossa of the occipital surface, and the load was 100 N) using Abaqus software.

**Results:**

The peak value and distribution of von Mises stress in each part of the experimental model were observed. After deep margin elevation, the peak dentin von Mises stresses were lower than the tensile strength of normal dentin in the post-core crown, endocrown, and inlay crown groups; the lowest stress results were found in the post-core crown group for the dentin, restoration, enamel, and deep margin elevation (DME) layers under vertical and oblique loading. In terms of stress distribution clouds, the peak stresses in the dentin tissue were located in the apical 1/3 of the root after postcore crown restorations for both loads, while stress concentrations were evident in the cervical and root areas after endocrown and inlay crown restorations; regardless of the load and restoration method, the corresponding stress concentration areas appeared at the junction of the DME and dentin tissue at the loading site of the restorations;

**Conclusions:**

Post-core crowns, endocrowns and inlay crowns can be used to restore residual crowns after deep margin elevation, and post-core crowns can better protect the residual tooth tissue.

## Introduction

Caries on the adjacent surface of posterior teeth are difficult to find due to their position. In these instances, the tooth always has extensive damage with pulpitis or apical periodontitis when evaluated by a dentist. When the adjacent caries edge is subgingival or below the cemento-enamel junction (CEJ), the complete shoulder is difficult to expose by conventional gingival drainage methods, and the existence of gingival crevicular fluid and saliva increases the difficulty of tooth preparation and impression preparation. This will further weaken the bonding strength of the indirect restoration [1]. To obtain a clean bonding interface and ensure the bonding effect, it is common to use crown lengthening [2], orthodontic traction [3] or a combination of both to obtain supragingival repair edges. Crown lengthening exposes the appropriate clinical crown height by properly removing gingival tissue and alveolar bone in accordance with the biological width. However, this technique needs to reduce the height of the periodontal supporting bone; thus, the treatment period is extended. In addition, the clinical efficacy is affected by anatomical factors of the affected teeth and technical factors such as bone excision, and root treatment [4]. Orthodontic traction refers to exposing the deep margin of a tooth defect by an orthodontic method [5]. This treatment has a long clinical operation time, and its feasibility and clinical effect are affected by many factors, such as the tooth crown-root ratio, root shape, root bifurcation and tooth position. Although these conventional treatments can obtain supragingival margins, they are not effectively applied in the clinic because of high cost, long repair time, large trauma, easy recurrence, difficulty being accepted by patients and other factors. To overcome the above defects, deep margin elevation was proposed as a new surgical method by Dietschi and Spreafico in 1998, and named “cervical marginal reconstruction” (CMR) [6]. In 2012, Magne and Spreafico called the same technique “deep margin elevation” (DME). Similar names, such as “coronal edge reconstruction (coronal margin relocation)” and “adjacent hole lifting (proximal box elevation) [7]”, can also be found in the literature. This technique suggests that the composite resin should be applied in the deepest part of the adjacent cavity to reposition the cervical edge above the gum, thus contributing to the placement of moisture barriers and rubber barriers, as well as improving the impression and adhesion of indirect restoration. In a sense, DME can be considered an alternative method to crown lengthening surgery. Compared with traditional repair methods, the deep margin elevation technique has many advantages, including less trauma, shorter repair time, easier acceptance by patients, and the ability to obtain the repair edge on the gingiva, which is convenient for the placement of rubber barriers and the subsequent bonding repair process. In addition, clinically, defects involving affected teeth under the CEJ require endodontic treatment, which can lead to a greater concentration of stress on the dentin when restoring high inlays in pulpless teeth compared to live-pulp teeth. Moreover, it has been confirmed that the type and thickness of the cushioning material in the pulpal cavity can affect the stress distribution and fracture resistance of the tooth when porcelain inlay restoration is performed on teeth after root canal treatment [8]. Therefore, when designing the deep margin elevation of root canal treated teeth, in addition to the type and height of the deep margin elevation material, the type and thickness of the pulpal cushioning material as well as the overall design of the gingival wall and pulpal cavity reconstruction may affect the stress distribution of the restorations and the dentin, which is an important factor to be considered in the design of the restorations [9]. So, the effect of this method on the stress distribution of restorations and teeth is still controversial, which makes many scholars carry out various studies on it.

In the past few decades, computational methods, especially finite element analysis (FEA), have become an indispensable methodology in the field of science and engineering and have been widely used to simulate real-life experiments, including industrial product development, machine design and biomedical research, especially in biomechanics and biomaterials [10]. With the continuous development of technology, the applicability of finite element analysis and modeling is improving. In the field of dentistry, this technique has become an important tool to analyze and predict the stress and strain of natural teeth, implants, restoration materials and surrounding bone tissues. Therefore, this study chose finite element analysis to flexibly establish residual crown models and different types of restoration models before and after deep margin elevation, applying vertical and oblique loads, comparing the stress distribution of each part of the experimental model, and then analyzing the effects of deep margin elevation on different restorations. The results are expected to provide biomechanical guidance for clinicians to select restorations.

## Materials and methods

### Materials and equipment

#### Experimental samples

One maxillary first premolar from a male adult patient was selected as the experimental sample. The tooth was required to have no obvious caries, wear, or tears. In addition, the morphology needed to conform to the standard of Chinese tooth morphology and anatomy. The patient voluntarily cooperated and signed the informed consent form based on full understanding. (Ethical Approval No. KYLL-2023-044)

#### Experimental equipment and software

A Lenovo notebook computer (IntelCorei7 dual-core processor, 64-bit operating system, memory capacity 8.0 GB hard disk capacity 1 TB CPU processing speed 2.5 GHz. Lenovo, Beijing); 3DX Multi-Image MicroCT (Morita, Japan); Mimics 17.0 (Materialise, Belgium); Geomagic studio 12.0 (Geomagic, USA); Hypermesh 12.0 (Altair, USA); Abaqus6.13 (SIMULIA, USA);

### Cone beam CT scanning to obtain sample data

The volunteer was told to open his or her mouth slightly, keeping a certain distance between the upper and lower dentition, and keeping the scanning section parallel to the orbital and ear plane as much as possible. The dentition and surrounding alveolar bone were horizontally scanned by 3D X Multi-Image Micro CT. The voltage was 80 kV, the resolution was 485 × 485, and the thickness and spacing of the layers were 0.125 mm. A total of 184 slices of tomographic images were obtained and stored in DICOM format.

### Establishing the residual crown model

**Fig. 1:**
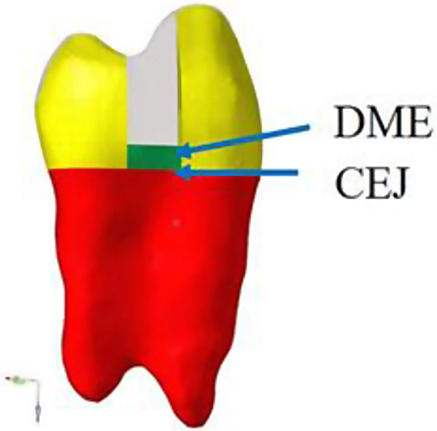
Model of maxillary first premolar residual crown: due to the recommended round preparation of the proximal box, the DME resin composite part is half-moon-shaped (green)

The DICOM data were imported into Mimics 17.0 medical imaging software, and various tissues in the file were identified and scanned. The right maxillary first premolar was extracted from the entire dentition, and its coronal, sagittal and horizontal images were automatically generated. After adjusting the image, the dentin, enamel and pulp cavities of the teeth were segmented in turn by setting different segmentation thresholds according to the gray differences between the enamel, dentin and pulp. The above images were masked, and then the software function was flexibly used and manually trimmed to improve the accuracy of the images. Finally, the 3D reconstruction function of the software was used to generate the original geometric model of the enamel, dentin and pulp of the maxillary first premolar. The models were exported to the STL format file and imported into Geomagic12.0 software. The original model of the maxillary first premolar was optimized and trimmed to remove the noise and sharp edges of the surface, fill the incomplete sections, and make the edges and surface of the model smooth by using the sanding, filling, cutting, smoothing, edge optimization and mesh doctor functions in Geomagic12.0 software. Then, using the software’s contour editing, surface patch construction and surface fitting functions, we accurately created the surface of the optimized trimmed model to produce a smooth, continuous and clear 3D model, and saved it (as shown in Fig. 1).

### Establishing the restoration model

The 3D model was imported into hypermesh software for refinement, and different mesh-densities were used for different parts of the tooth according to the complexity of the model geometry, producing a model of the maxillary first premolar with enamel, dentin and pulp cavity. The dentin on the root surface was expanded 0.2 mm outward to form the periodontium, which contacts the lateral alveolar bone. The original model was expanded, sheared, fitted and Booleaned to produce a model of the subgingival stump, enamel, prepared dentin, DME, fiber post core, resin core, pulp, cementum cusp, periodontal membrane and alveolar bone. With reference to clinical standards for restoration preparation, an experimental group model of the residual crown before and after deep margin elevation, restored by a post core crown, an endocrown and an inlay crown, was constructed. The design points for each restoration are as follows:

#### Design of the post core crown group

**Fig. 2:**
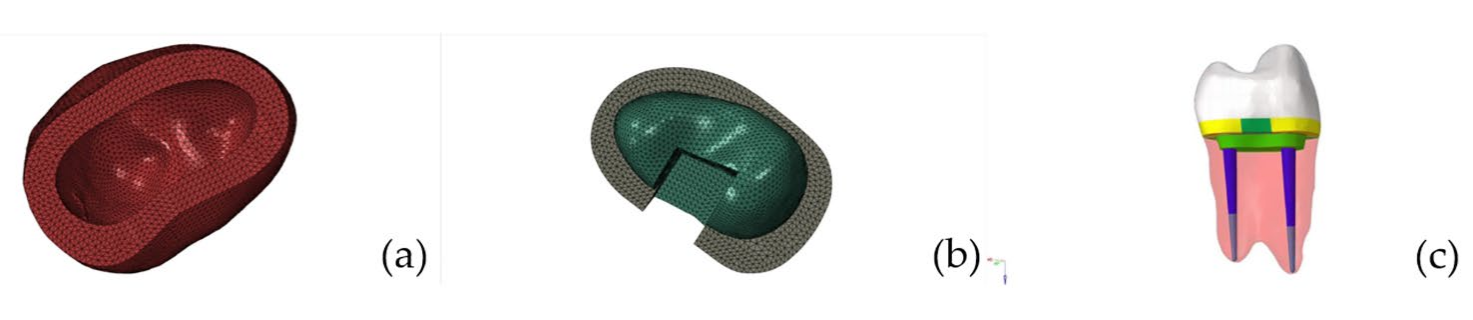
Experimental model of the post-core crown group: (**a**) restoration; (**b**) dentin; (**c**) tooth model

The post core crown group was set up as a fiber post and crown with a resin core. The diameter of the post in the root canal was 1/3 of the root canal diameter, the length of the post was 2/3 of the root length, and the 4 mm apical sealing zone was preserved. The crown was prepared to simulate a full crown with an axial taper of 5° and a crown margin with a right-angled shoulder of 1 mm in width. The surface of the preparation was rounded (Fig. 2).

#### Design of pulp endocrown group

**Fig. 3:**
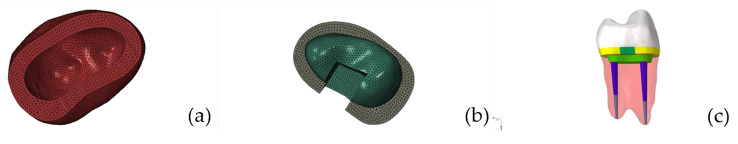
Experimental model of the endocrown group: (**a**) restoration; (**b**) dentin; (**c**) tooth model

The margins of the pulp chamber crown group were in the form of a butt-joint edge. The depth of the pulp chamber was at least 3 mm, and the bottom surface of the pulp chamber was covered with a 1 mm thick cushioning material. The remaining peripheral axial wall was at least 1 mm thick, and the pulp chamber was extended 5° by removing the intracavity recess. The surface of the preparation was rounded at the point line, (see Fig. 3).

#### Design of the inlay crown group

**Fig. 4:**
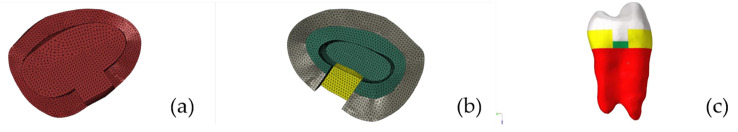
Experimental model of the inlay crown group: (**a**) restoration; (**b**) dentin; (**c**) tooth model

The inlay crown group was prepared in the pulp cavity as in the pulp endocrown group, and the extra coronal preparation was prepared as in the post core crown group. The margin was a right-angled shoulder of 1 mm width, with a rounded dotted line on the surface of the preparation (see Fig. 4).

### Material assignment

All materials were assumed to be homogeneous, continuous and isotropic elastic linear materials [11, 12]. The material properties of each component are given in Table 1.

**Table 1:** Material properties

Material	Elastic modulus/Gpa	Poisson’s ratio
Enamel	84.1	0.33
Dentin	18.6	0.31
Cortical bone	13.7	030
Alveolar bone	1.37	0.30
Periodontal ligament	0.05	0.49
Flowable resin	6.50	0.30
Gutta percha	6.9*10 − 4	0.45
Indirect ceramic (Emax)	95	0.32
Resin core	14.1	0.24
Fiberglass post	37	0.34

### Loading and boundary conditions

**Fig. 5:**
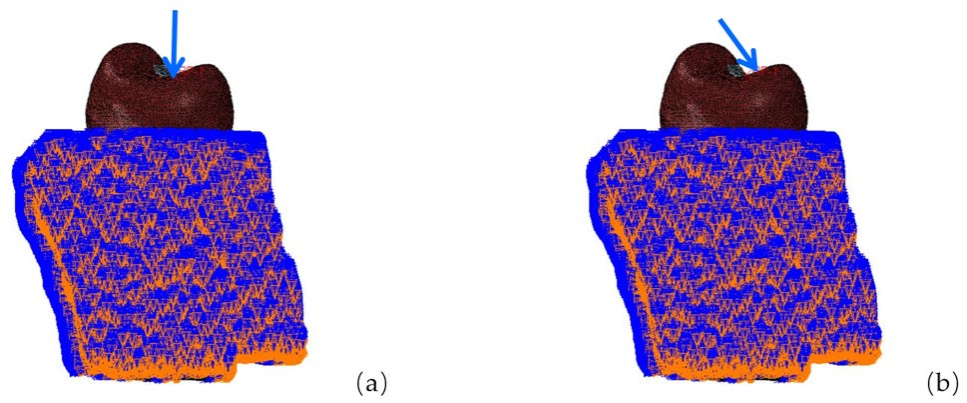
Stress load: (**a**) vertical load ; (**b**) oblique load

Maxillary first premolars are mainly subjected to vertical forces from parallel to the long axis of the tooth and lateral forces from the palatal side [13, 14]. Therefore, the loading direction was set at 45° to the long axis of the tooth or parallel to the long axis of the tooth. A static constant homogeneous force of 100 N was used to simulate the maximum load during normal occlusion of the maxillary first premolar, fixing the base of the alveolar bone [15, 16]. The loading position was approximately 1.5 mm × 2 mm in the central fossa of the occlusal surface. The loading mode is shown in Fig. 5.

### Observation indicator

Von Mises was chosen as an indicator for stress analysis. It is a common indicator for predicting-the fatigue and damage potential of materials [17]. According to the fourth strength theory in engineering mechanics, regardless of the conditions of stress, when the morphological change ratio at a point within the material reaches the unidirectional limit boundary, the material undergoes plastic yielding damage, and a larger stress value represents a greater chance of yielding damage [18].

## Results

### Stress analysis of different parts of the experimental groups after deep margin elevation

#### Peak Von Mises stresses in different parts of the model for each group

**Fig. 6:**
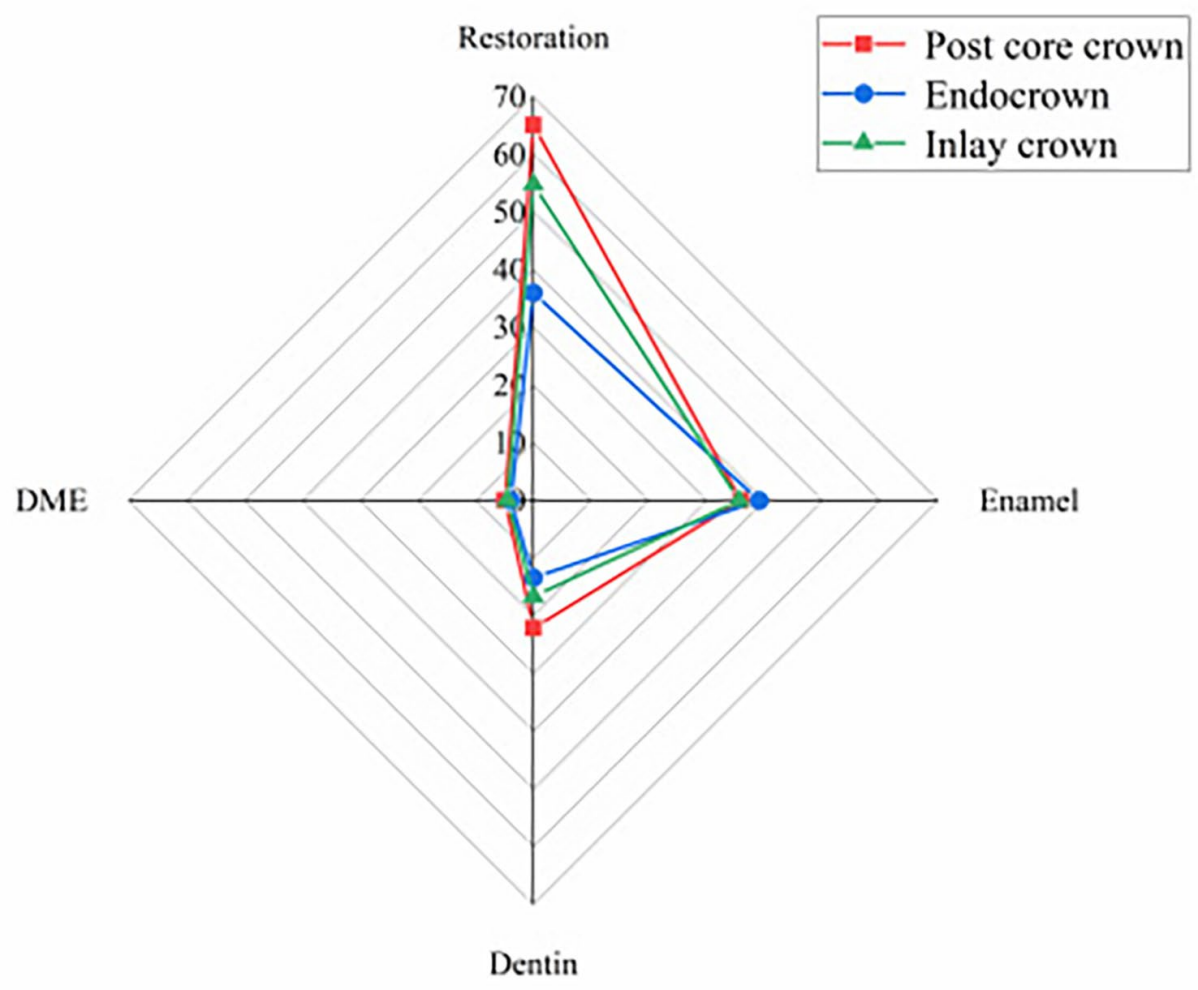
Peak von Mises stress in each part of the repair under vertical load

**Fig. 7:**
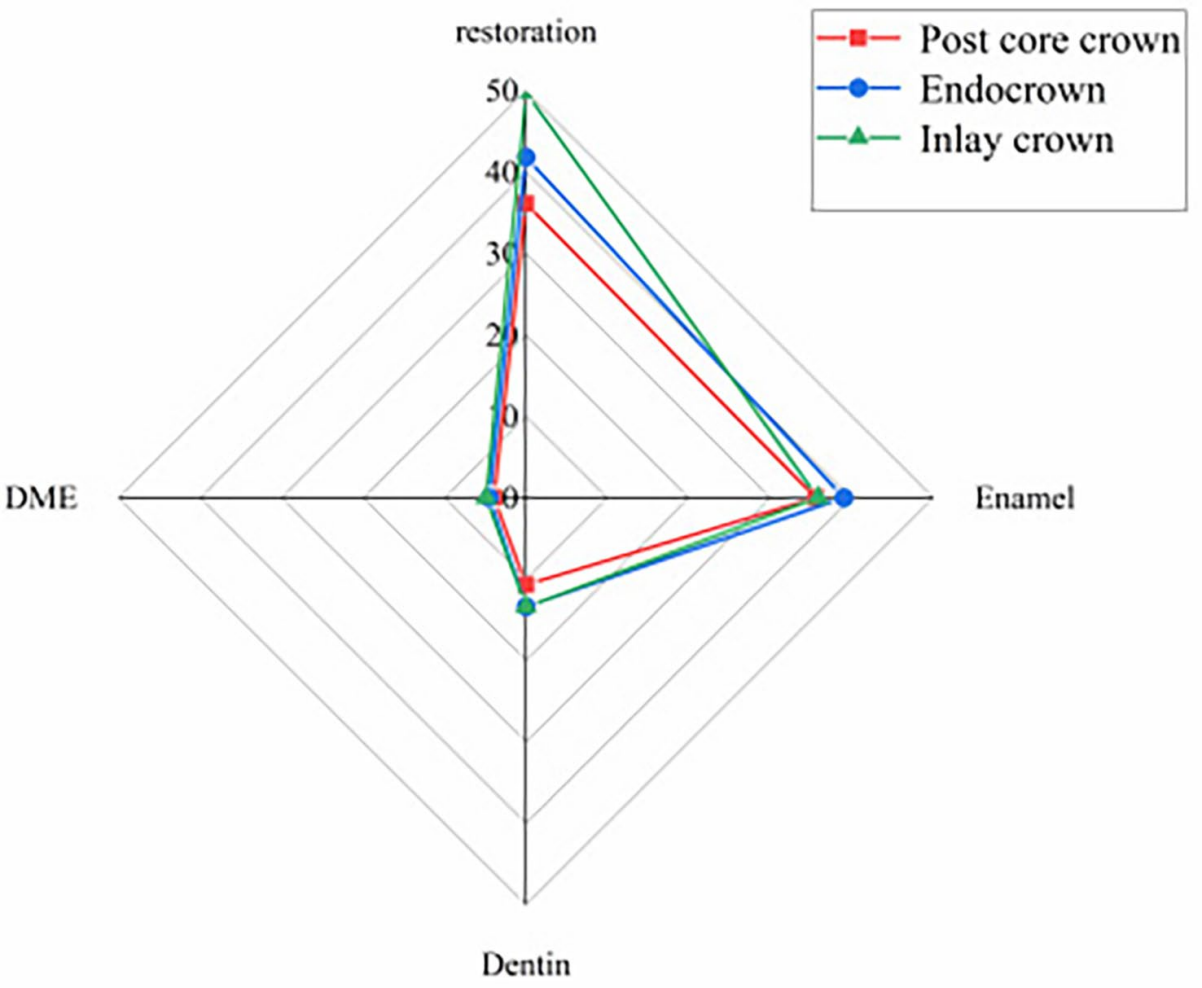
Peak von Mises stress in various parts of the repair under oblique load

The results of the peak von Mises stresses in each part of a residual crown after deep margin elevation, restored by different methods, under vertical and oblique loads are shown in Figs. 6 and 7.

#### Magnitude and distribution of dentin equivalent forces under two kinds of loads

**Fig. 8:**
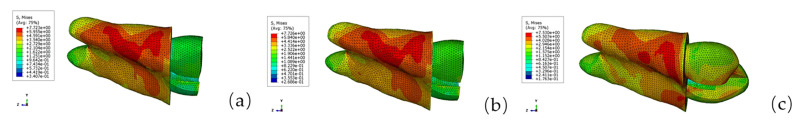
Cloud of stress distribution in the dentin layer of different restorations under vertical loading: (**a**) endocrown; (**b**); inlay crown; (**c**) post core crown

**Fig. 9:**
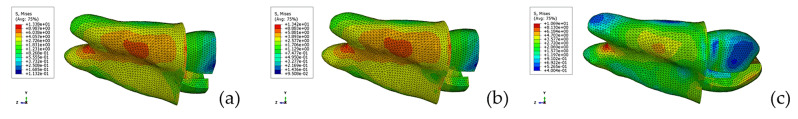
Cloud of stress distribution in the dentin layer of different restorations under oblique loading: (**a**) endocrown ; (**b**) ; inlay crown; (**c**) post core crown

**Fig. 10:**
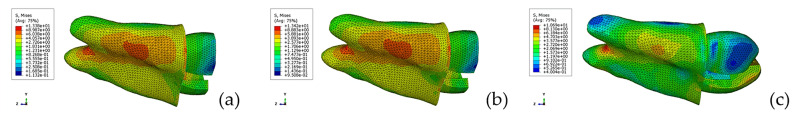
Stress distribution clouds of restorations with different restoration methods under vertical loading: (**a**) endocrown; (**b**); inlay crown; (**c**) post core crown

Under vertical load, the dentin stress in the post-core crown, endocrown and inlay crown groups was mainly concentrated in the cervical and root regions of the tooth. The peak stress of the post core crown group was located in the apical 1/3 of the buccolingual root in contact with the post, and the peak stress in the endocrown group was located between the bottom of the pulp chamber and the palatal root canal orifice. The peak stress in the inlay crown group was located in the dental cervix (see Fig. 8). Under oblique loading, the peak stress of the post core crown group was at the same location as the vertical load, in the area of contact between the root canal and the post; the peak stress in the endocrown group was concentrated in the cervical region, with the same peak stress as the vertical load; and the peak stress in the inlay crown group was located at the bifurcation of the buccal and lingual roots (see Fig. 9). The peak equivalent stress in the dentin of each model group under two types of loading is shown in Figs. 9 and 10. The peak stresses in the dentin of the post-core crown group were the lowest for vertical loading, with a decrease of 2.46% (7.72–7.53 MPa) compared to the endocrown group, and 2.59% (7.73–7.53 MPa) compared to the inlay crown group. The peak dentin stresses were also lowest in the post-core crown group during oblique loading, with a reduction of 20.10% (13.38–10.69 MPa) compared to the endocrown group and 20.34% (13.42–10.69 MPa) compared to the inlay crown group.

#### Magnitude and distribution of Von Mises in different restorations under two loads

**Fig. 11:**
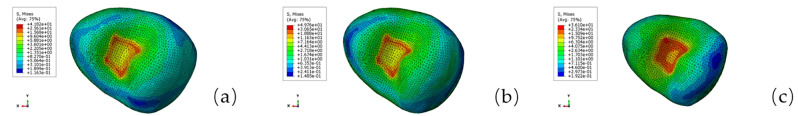
Stress distribution clouds of restorations with different restoration methods under oblique loading: (**a**) endocrown; (**b**); inlay crown; (**c**) post core crown

Under different loading conditions, the peak von Mises stresses within the post-core crown, inlay crown and endocrown restorations all occurred at the loading site, with significant stress concentrations (see Figs. 10 and 11). During vertical loading, the peak stresses within the post core crown restoration were the lowest, with the endocrown group increasing by 25.70% (39.96–31.79 MPa) compared to the post core crown group, while the inlay crown group increased by 46.52% (46.58–31.79 MPa) compared to the post core crown group. The peak stresses within the post core crown restoration were also lowest during oblique loading, with 15.84% (41.82–36.10 MPa) higher in the medullary cemented crown compared to the post core crown group and 37.84% (49.76–36.10 MPa) higher in the inlay crown group, (see Figs. 6 and 7).

#### Magnitude and distribution of enamel Von Mises under two types of loading

**Fig. 12:**
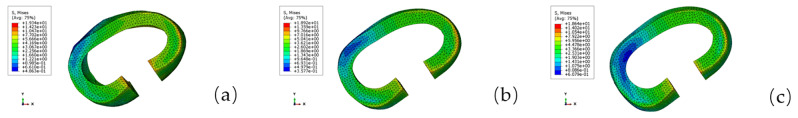
Stress distribution clouds of the enamel layer for different restorations under vertical loading: (**a**) endocrown ; (**b**) ; inlay crown; (**c**) post core crown

**Fig. 13:**
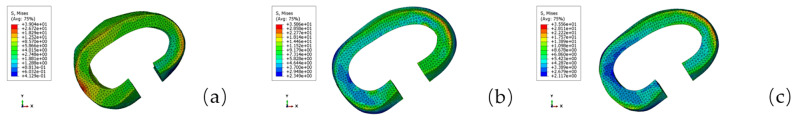
Cloud of stress distribution in the enamel layer of different restorations under oblique loading: (**a**) endocrown ; (**b**) ; inlay crown; (**c**) post core crown

Under vertical loading, the peak stress of the pile core crown group was located at the lingual enamel-dentin junction; the peak stress of the pulpal retention crown group was located at the enamel-dentin junction adjacent to the buccal cavity margin; and the peak stress of the inlay crown group was located at the lingual enamel-dentin junction (see Fig. 12). Under oblique loading, the peak stress site of the pile crown group was the same as that of the vertical loading; the peak stress of the pulpal retention crown group was located at the junction of the enamel dentin on the buccal side; and the peak stress of the inlay crown group was the same as that of the vertical loading. (see Fig. 13).

The peak von Mistresses in the enamel of each model group under both loads are shown in Figs. 6 and 7. The lowest peak enamel stresses for vertical loading were observed in the post core crown group, which decreased by 3.62% (19.34–18.64 MPa) compared to the inlay crown group and by 1.48% (18.92–18.64 MPa) compared to the pulpal-cemented crown group. The peak enamel stresses were also lowest in the post core crown group when loaded obliquely, with a reduction of 8.91% (39.04–35.56 MPa) compared to the inlay crown group and 0.84% (35.86–35.56 MPa) compared to the endocrown group.

#### Magnitude and distribution of DME Von Mises under two loads

**Fig. 14:**
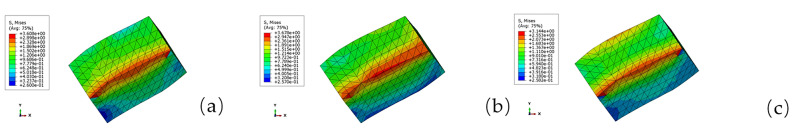
Stress distribution clouds of DME layers with different repair methods under vertical load: (**a**) endocrown; (**b**); inlay crown; (**c**) post core crown

**Fig. 15:**
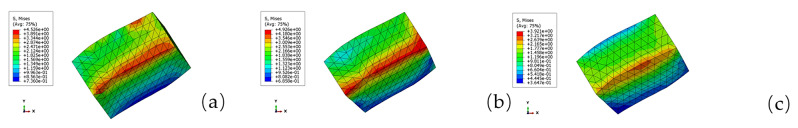
Stress distribution clouds of DME layers with different repair methods under oblique load: (**a**) endocrown ; (**b**) ; inlay crown; (**c**) post core crown

Under different loading conditions, the DME stress distribution in the post core, endocrown and inlay crowns were all mainly concentrated at the corner of the lingual cavity margin and the deep margin (see Figs. 14 and 15). During vertical loading, the peak stresses were lowest in the post core crown group, being 14.67% (3.68–3.14 MPa) lower than that in the inlay crown group and 13.02% (3.61–3.14 MPa) lower than that in the endocrown group. The peak stresses were also significantly lower in the post core crown group than in the other two groups during oblique loading, decreasing by 13.47% (4.53–3.92 MPa) and 20.49% (4.93–3.92 MPa) compared to the endocrown and inlay crown groups, respectively (see Figs. 6 and 7).

There were a total of 16 nodes at the edge of the interface between the DME and the dental tissue. From these, 8 nodes were randomly selected to test the stress distribution: 1 (252,396), 2 (248,149), 3 (248,147), 4 (248,145), 5 (248,143), 6 (248,141), 7 (248,139) and 8 (248,081).

**Fig. 16:**
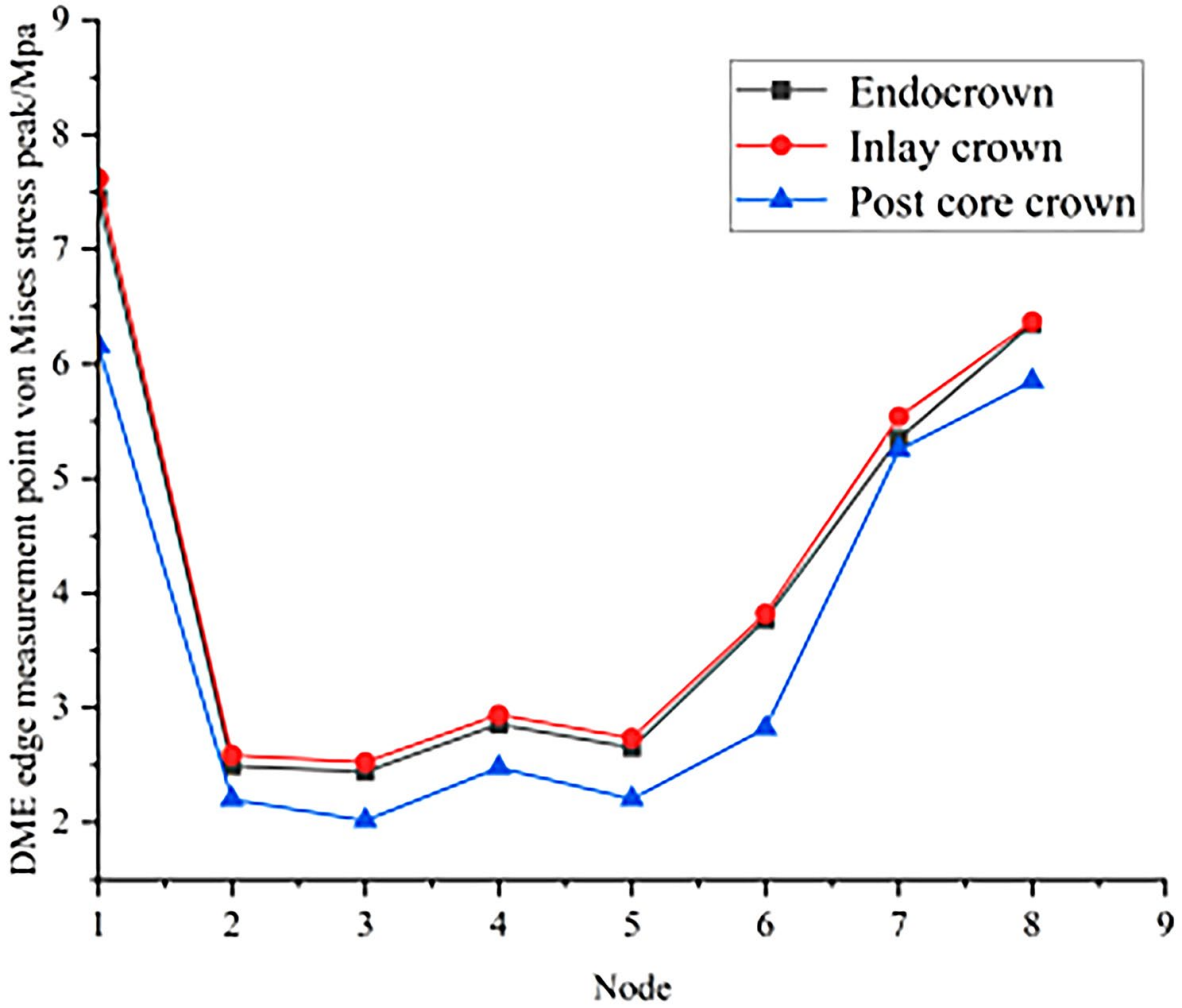
Peak von Mises stress at DME edge measurement points for different repair methods under vertical load

**Fig. 17:**
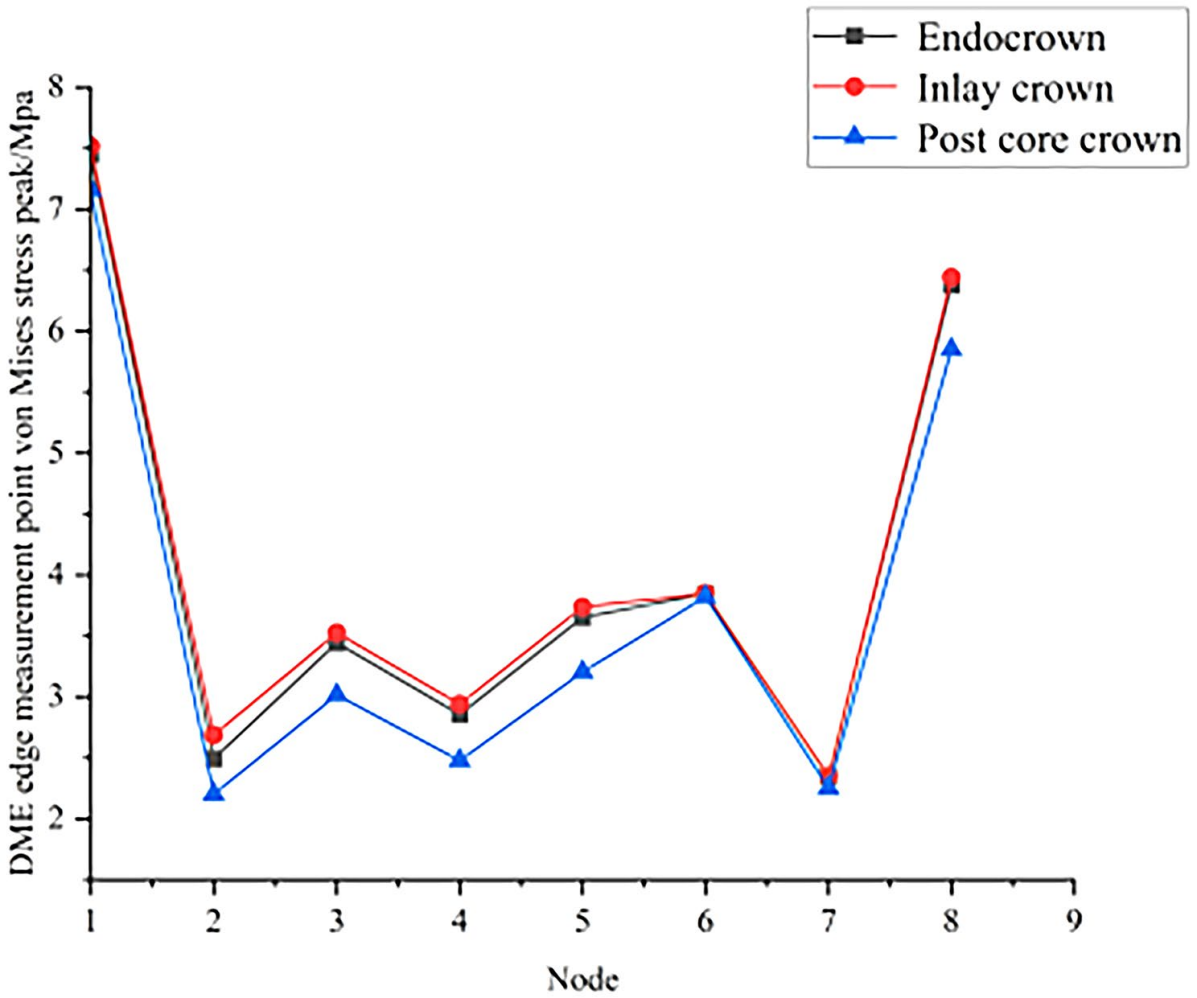
Peak von Mises stress at DME edge measurement points for different repair methods under oblique load

As seen in Figs. 16 and 17, the peak stresses at the contact sites between the DME and the margins of the tooth were all post core crowns < endocrowns < inlay crowns under vertical and oblique loading.

## Discussion

### Selection of finite element analysis and setting of stress loading conditions

In the past few decades, computational methods, especially FEA, have become an indispensable methodology in the field of science and engineering and have been widely used to simulate real-life experiments, including industrial product development, machine design and biomedical research, especially in biomechanics and biomaterials [19]. With the continuous improvement of technology, the applicability of finite element analysis and modeling is also improving. In the field of stomatology, this technique has become an important tool for analyzing and predicting the stress and strain of natural teeth, implants, restoration materials and surrounding bone tissue [20]. Therefore, in this study, finite element analysis was chosen to flexibly establish the residual crown model and different types of restoration models, before and after deep margin elevation, to which both vertical and oblique loads were applied, and the stress distribution in each part of the experimental model was compared. Finally, a comprehensive analysis of the effects of deep margin elevation on different restorative methods was carried out to provide theoretical guidance to clinicians in the selection of restorations.

It has been shown that when using the finite element method to simulate intraoral masticatory stresses in restorations, the results of loading static forces are consistent with those of loading dynamic forces, with the former being an alternative to the latter. Therefore, in this study, static force loading was chosen for stress loading of the restorations [21]. In addition, with reference to the relevant literature, the vertical load parallel to the long axis of the tooth and the oblique load at an angle of 45° with the long axis of the tooth were applied to the central fossa of the restoration occlusal face to simulate the force on the tooth during lateral occlusion and intercuspal occlusion.

Previous studies have shown that [22], the maximum bite force that premolars can withstand is 600–700 N, while the average bite force during mastication is only 30-40% of the maximum bite force. Therefore, in this study, both vertical and oblique loads were set to 100 N to simulate the force during normal mastication.

### On the choice of restoration form

In Class II cavities with large adjacent surface defects, especially residual crowns after deep margin elevation, inlay and onlay restorations can result in greater stress concentration on the dentin, making the dentin tissue more susceptible to fracture [23]. Therefore, a post core or inlay crown is often used to restore a large defect on the adjacent surface of a tooth. Post core crowns add additional retention to the crown restoration by placing a post in the root canal, while post core crowns [24] facilitate the rootward transmission of forces, but the preparation process can increase the degree of defect and may lead to irreversible root fracture. The inlay crown is based on a full crown preparation using an inlay that extends into the pulp cavity to provide secondary retention. The internal and external mechanical forces and the locking effect between the full crown and the inlay crown can significantly improve the retention of the restoration. However, extracoronal and intracoronal dentin preparation significantly damages healthy cervical dentin, weakening the fracture resistance of the restored dentin and further increasing the risk of fracture, contrary to the theory of protecting peri-cervical dentin [25]. On this basis, the endocrown was developed. The cervical dentin is maximally preserved by the butt-joint edge preparation of endocrowns, which use the wide pulpal cavity of the molar after root canal treatment as a central retentive form and dentin bonding to provide retention [26]. However, some clinicians are skeptical about the effectiveness of this retention method, as it is not consistent with the concept of creating a macroscopic mechanical retention pattern in traditional restoration. In summary, there are advantages and disadvantages to the use of postcore crowns, inlay crowns and endocrowns as common treatment modalities for restoring residual crowns; thus, these three restorations were chosen as the experimental groups to restore residual crowns before and after deep margin elevation.

### Discussion of experimental results

#### Comparison of stress distribution of different restorations after deep margin elevation

In terms of the dentin von Mises stress cloud and peak values, the peak dentin stresses for vertical and inclined loading were inlay crowns > endocrowns > post core crowns. This shows that the post-core crown restoration bears a lower occlusal force on the dentin tissue, which is more favorable to the protection of the dentin tissue. This may be related to the fact that the post-core crown, which has a high modulus of elasticity, bears more of the occlusal force, allowing the stress to be distributed more evenly in the dentin, thus avoiding higher stresses in the local area [27]. The stress distribution cloud showed that the stress distribution was more dispersed in post-core crowns than in endocrowns and inlay crowns under vertical and oblique loading, suggesting that more healthy dentin is removed during the preparation of inlay and pulpal crowns, which is not conducive to stress dispersion and rootward transmission [28]. The peak stress of the endocrown was located at the base of the pulp chamber and at the palatal root canal interval under different loads, which suggests that the cusp slope of the palatal cusp should be decreased to reduce the lateral forces on it and that the smooth and rounded edges of the base of the pulp chamber should be taken into account during the preparation of the tooth to avoid creating a stress concentration zone. In the inlay crown group, the peak stress is located in the cervical region during vertical loading, which may be associated with the reduction of enamel in this region, while during oblique loading, the stress is relatively dispersed, which can better protect the dental tissues. This is generally consistent with the findings of Zarow et al. [29].

The peak von Mises forces for the restorations of the postcore crown, endocrown and inlay crowns in this test were all found in the loading region, where there were significant stress concentrations, as shown in other finite element studies [30]. This indicates that the restorations are susceptible to damage in the stress loading region, implying the need to ensure a certain thickness of the porcelain layer to resist occlusal forces in clinical practice. It was also observed that the peak stresses in the restorations were significantly increased in all groups when loaded obliquely compared with when loaded vertically, which suggests that the lateral forces generated during mastication increase the burden on the dental tissues and result in a larger possibility of damage, especially when restoring teeth that are subjected to more lateral forces during mastication, such as maxillary premolars, which must reduce the damage caused by lateral forces [31].

Analysis of the peak stresses in the enamel in each group after deep margin elevation showed that the peak stresses in the enamel during vertical and inclined loading were endocrowns > inlay crowns > post core crowns, which may be related to the fact that the endocrown group had significantly more enamel remaining at the time of preparation than the other two groups. Enamel has a higher modulus of elasticity and therefore serves to support restoration. The more enamel remaining, the more stress it is subjected to and the higher the peak stresses, which is in general agreement with the results of the mechanical study by Mangold and Kern on mandibular premolars [32]. The stress distribution cloud showed that the cervical areas of the different restorations were areas of high stress concentration, which is characteristic of the stress distribution phenomenon in natural teeth. This is mainly dependent on factors such as the shape of the tooth and its structure. The maxillary premolar is obviously narrowed in the cervical region, which leads to a transitional area of shape where stresses are more concentrated. There is also a portion of enamel in the cervical region, and the enamel at the enamel-dentin boundary forms a sharp edge, resulting in a concentration of stress. Enamel plays an important role in the transmission and dispersion of forces, resulting in the formation of stress concentration areas near the enamel margins in the cervical region, which are the force-bearing tissues of the tooth [33].

From the analysis of the stress distribution of the deep margin elevation material, regarding less of the loading conditions and the restoration method, the stressconcentration area appeared at the corner of the lingual cavity margin and the gingival wall, suggesting that attention should be given to rounding the corner of the point line here to reduce stress concentration during a clinical operation, which may be related to the high volume shrinkage of the flowable composite resin material that can contribute to high stress values, a result that is consistent with the findings of Zhang [34], Baldi [35] and others who have studied the mechanics of maxillary premolar teeth. However, this result is not consistent with the study by Jurado C A et al [36]. because the study was performed on mandibular premolars, which do not have the same anatomical morphology as maxillary premolars, and the maximum reduction in cervical height in this experiment was 2 mm below the enamel-cementum boundary. The peak stresses generated by the DMEs during vertical and oblique loading, however, did not exceed their own tensile and compressive strengths, indicating that the DMEs have little probability of fracture under normal occlusal forces, but the long-term effects are unknown, a result that is generally consistent with the study by Chen et al. [37]. In addition, the peak stresses of the post-core crown group were significantly lower than those of the other two groups under different loading conditions, which may be correlated with the fact that postcore crowns are more protective of *the* dental tissue.

### Innovativeness and deficiency

The deficiency in this study is the omission of the bonding layer mesh from the finite element model. This may affect the performance of the restoration, as there has been some literature showing a correlation between the mechanical properties of the restoration and the bonding agent [35]. However, at present, it is difficult to obtain realistic and accurate bond layer meshes that can be easily transformed in a finite element model due to the thickness and material properties of the bond layer. Removing the bond layer removes the need to consider preprocessing and solution time, speeding up calculations and avoiding the introduction of potential errors in this critical region. Furthermore, despite the realistic anisotropy of dentin and enamel, all materials were simulated using the uniform isotropy law. Due to time constraints, relevant in vitro experiments could not be performed to further verify the accuracy of the experimental results. Therefore, in subsequent experiments, the team will continue to improve the experimental design and complete the relevant contents as much as possible to enhance the reliability of the experimental results.

## Conclusions

Post-core crowns, endocrowns and inlay crowns can all be used for the restoration of residual crowns after deep margin elevations, with postcore crowns being a preferable option because they provide both better protection of the remaining tooth tissue and a stable retention effect.

## Data Availability

All data generated or analysed during this study are included in this published article.
